# Cholangioscope-assisted evaluation and endoscopic incision of stricture caused by thick mucosal bridge in Crohn’s disease

**DOI:** 10.1055/a-2374-8662

**Published:** 2024-08-16

**Authors:** Xianzong Ma, Lang Yang, Yan Jia, Zilin Kang, Mingjie Zhang, Dongliang Yu, Peng Jin

**Affiliations:** 1 Department of Gastroenterology, The Seventh Medical Center of Chinese PLA General Hospital, Beijing, China; 2 104607 Chinese PLA General Hospital, Beijing, China; 3 Senior Department of Gastroenterology, The First Medical Center of Chinese PLA General Hospital, Beijing, China

A 32-year-old man with an 8-year history of Crohn’s disease (Montreal classification A2, L3, B2p) presented with recurrent abdominal pain. After regular treatment with ustekinumab for 3 years, recent computed tomography revealed severe stricture in the ascending colon ( Fig. 1 ), and colonoscopy showed colorectal mucosal healing. However, two tiny holes were observed and could not be passed by the colonoscope ( Fig. 2 ).

**Fig. 1:**
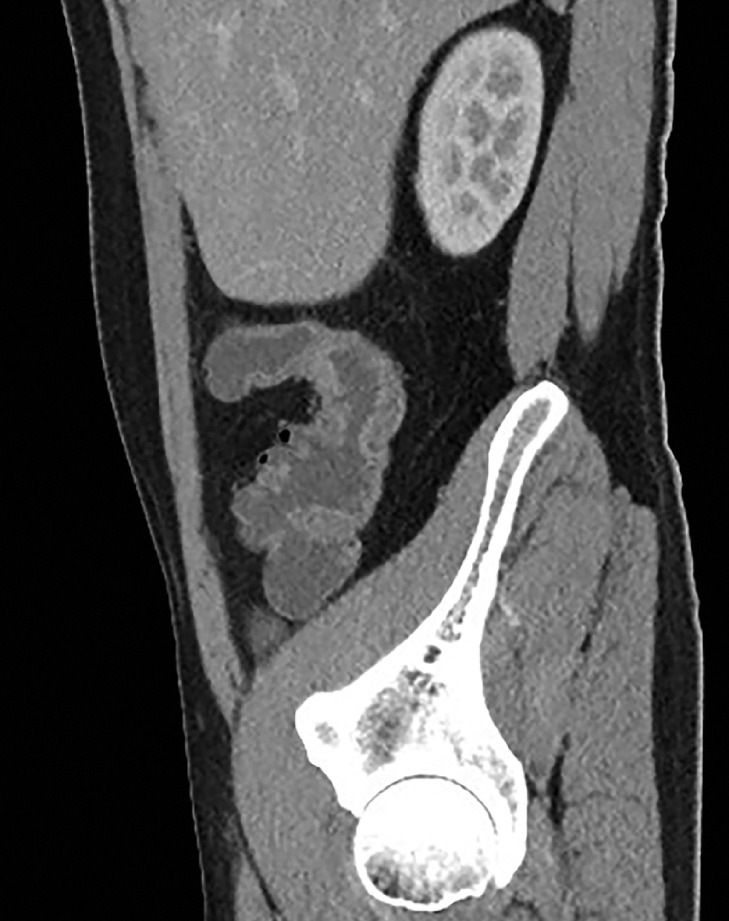
Computed tomography showed severe stricture of the ascending colon in Crohn’s disease.

**Fig. 2:**
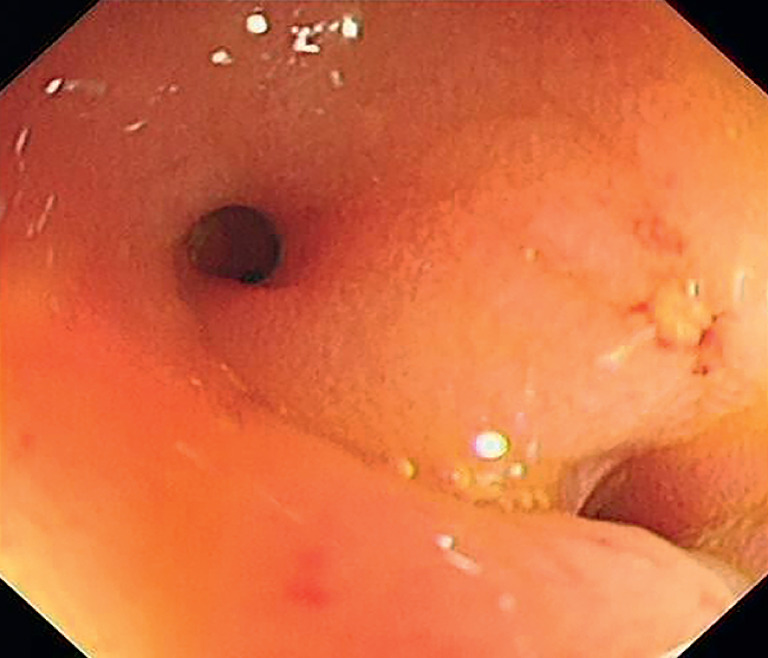
Two tiny holes in the stricture were observed and could not be passed by the colonoscope.

In order to find out which hole was the real narrow bowel lumen and to exclude a potential fistula, a cholangioscope (eyeMAX, 9 F; Micro-Tech, Nanjing, China) was inserted into the two holes respectively to gain direct views inside and behind the holes ( Fig. 3 ) 1 2 . Amazingly, the ileocecal valve was reached by the cholangioscope through both holes, and superficial ulcer in the inner wall of the holes and multiple scar changes in the ileocecal region were observed simultaneously ( Fig. 4 ). The cholangioscopy result indicated that the stricture was caused by a rare thick mucosal bridge between the two holes. Subsequently, the bridge mucosa and submucosal scar were incised by an ITknife nano (Olympus, Tokyo, Japan) ( Fig. 5 ). No active bleeding or perforation occurred during the procedure. Finally, the colonoscope could pass smoothly through the stricture and reach the ileocecal valve ( Video 1 ).

**Fig. 3:**
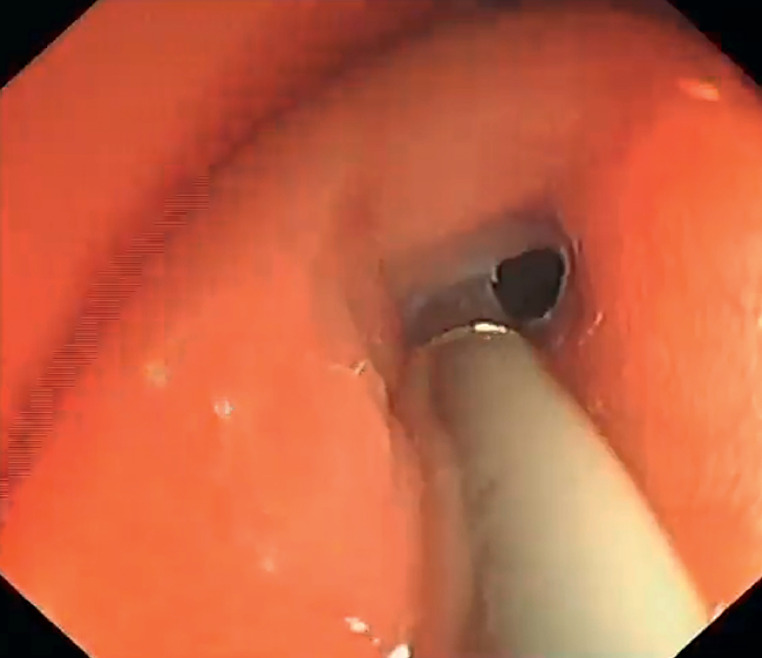
The cholangioscope was inserted into one hole.

**Fig. 4:**
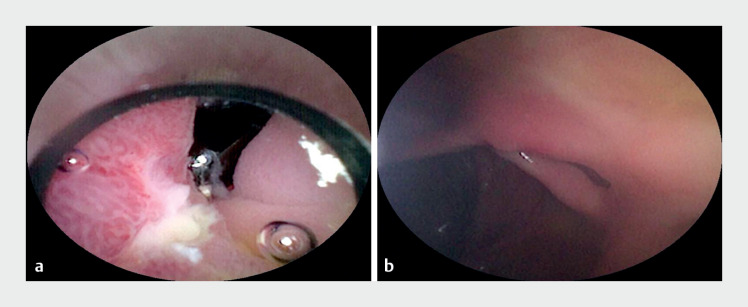
Cholangioscopy showed superficial ulcers in the inner wall of the stricture. The cholangioscope reached the ileocecal valve through both holes.

**Fig. 5:**
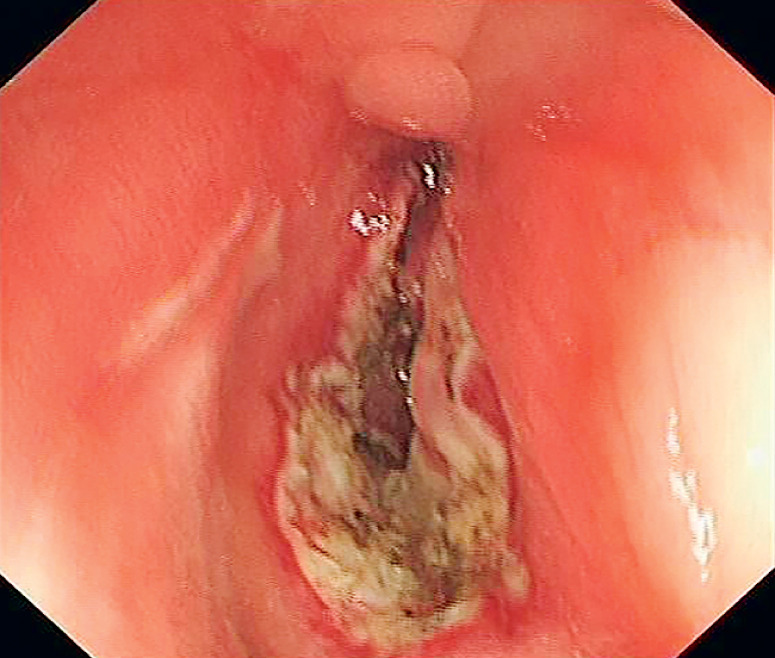
The stricture was treated after incision of the bridge mucosa and submucosal scar under colonoscopy.

Cholangioscopy-assisted evaluation and targeted treatment for Crohn’s disease-associated stricture.Video 1

Currently, the evaluation of bowel stenosis in Crohn’s disease is mainly based on radiology and ultrasonography 3 . However, neither method can directly reveal the clear presentation of the inner wall of the narrowed intestinal lumen, including ulcers, tiny fistula, and edema near the stricture. To our knowledge, this is the first reported case of cholangioscope-assisted evaluation and management of Crohn’s disease-related stricture, and suggests the feasibility of the procedure in selected Crohn’s disease cases.

Endoscopy_UCTN_Code_TTT_1AQ_2AF

## Notes

Endoscopy E-Videos https://eref.thieme.de/e-videos
*E-Videos* is an open access online section of the journal *Endoscopy* , reporting on interesting cases and new techniques in gastroenterological endoscopy. All papers include a high-quality video and are published with a Creative Commons CC-BY license. Endoscopy E-Videos qualify for HINARI discounts and waivers and eligibility is automatically checked during the submission process. We grant 100% waivers to articles whose corresponding authors are based in Group A countries and 50% waivers to those who are based in Group B countries as classified by Research4Life (see: https://www.research4life.org/access/eligibility/ ). This section has its own submission website at https://mc.manuscriptcentral.com/e-videos .
